# Enhancement of thioredoxin/glutaredoxin-mediated L-cysteine synthesis from *S*-sulfocysteine increases L-cysteine production in *Escherichia coli*

**DOI:** 10.1186/1475-2859-11-62

**Published:** 2012-05-18

**Authors:** Takeshi Nakatani, Iwao Ohtsu, Gen Nonaka, Natthawut Wiriyathanawudhiwong, Susumu Morigasaki, Hiroshi Takagi

**Affiliations:** 1 Graduate School of Biological Sciences, Nara Institute of Science and Technology, 8916-5 Takayama, Ikoma, Nara 630-0192, Japan; 2 Research Institute for Bioscience Products and Fine Chemicals, Ajinomoto Co., 1-1 Suzukicho, Kawasaki, Kanagawa 210-8681, Japan; 3 National Center for Genetic Engineering and Biotechnology, National Science and Technology Development Agency, 113 Thailand Science Park, Phahonyothin Road, Klong 1, Klong Luang, Pathumthani 12120, Thailand

**Keywords:** Thiosulfate pathway, L-cysteine, Redox enzyme, Sulfite reductase, *S*-sulfocysteine

## Abstract

**Background:**

*Escherichia coli* has two L-cysteine biosynthetic pathways; one is synthesized from *O*-acetyl L-serine (OAS) and sulfate by L-cysteine synthase (CysK), and another is produced via *S*-sulfocysteine (SSC) from OAS and thiosulfate by SSC synthase (CysM). SSC is converted into L-cysteine and sulfite by an uncharacterized reaction. As thioredoxins (Trx1 and Trx2) and glutaredoxins (Grx1, Grx2, Grx3, Grx4, and NrdH) are known as reductases of peptidyl disulfides, overexpression of such reductases might be a good way for improving L-cysteine production to accelerate the reduction of SSC in *E. coli*.

**Results:**

Because the redox enzymes can reduce the disulfide that forms on proteins, we first tested whether these enzymes catalyze the reduction of SSC to L-cysteine. All His-tagged recombinant enzymes, except for Grx4, efficiently convert SSC into L-cysteine *in vitro*. Overexpression of Grx1 and NrdH enhanced a 15-40% increase in the *E. coli*L-cysteine production. On the other hand, disruption of the *cysM* gene cancelled the effect caused by the overexpression of Grx1 and NrdH, suggesting that its improvement was due to the efficient reduction of SSC under the fermentative conditions. Moreover, L-cysteine production in knockout mutants of the sulfite reductase genes (Δ*cysI* and Δ*cysJ*) and the L-cysteine synthase gene (Δ*cysK*) each decreased to about 50% of that in the wild-type strain. Interestingly, there was no significant difference in L-cysteine production between wild-type strain and gene deletion mutant of the upstream pathway of sulfite (Δ*cysC* or Δ*cysH*). These results indicate that sulfite generated from the SSC reduction is available as the sulfur source to produce additional L-cysteine molecule. It was finally found that in the *E. coli*L-cysteine producer that co-overexpress glutaredoxin (NrdH), sulfite reductase (CysI), and L-cysteine synthase (CysK), there was the highest amount of L-cysteine produced per cell.

**Conclusions:**

In this work, we showed that Grx1 and NrdH reduce SSC to L-cysteine, and the generated sulfite is then utilized as the sulfur source to produce additional L-cysteine molecule through the sulfate pathway in *E. coli*. We also found that co-overexpression of NrdH, CysI, and CysK increases L-cysteine production. Our results propose that the enhancement of thioredoxin/glutaredoxin-mediated L-cysteine synthesis from SSC is a novel method for improvement of L-cysteine production.

## Background

L-Cysteine is the most important sulfur-containing organic compound, and it is required for the biosynthesis of sulfur-containing compounds such as l-methionine, thiamine, biotin, and coenzymes A. In addition, L-cysteine plays crucial roles in protein folding, assembly, and stability through disulfide-bond formation. L-Cysteine-containing proteins, such as thioredoxin (Trx) and glutaredoxin (Grx), are involved in protecting cells under oxidative stress conditions. Recently, we have proposed that the periplasmic L-cysteine protects *E. coli* cells from hydrogen peroxide, which is produced by phagocytes, in the environment [1]. Since L-cysteine has the essential functions in cellular metabolism, it is also an important amino acid in terms of its applications in the pharmaceutical, food, and cosmetic industries.

**Figure 1:**
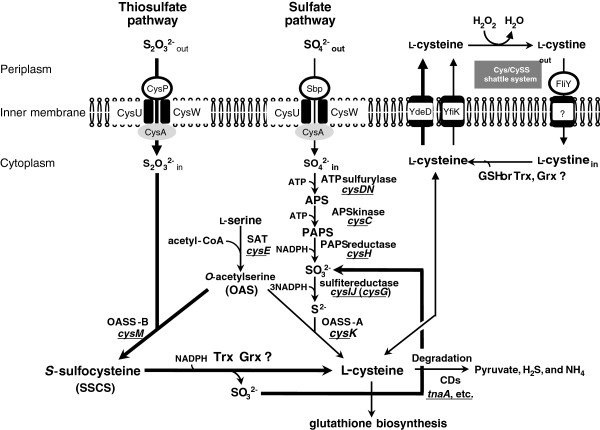
**Sulfur assimilation and L-cysteine synthetic pathway in *Escherichia coli. ****E. coli* has sulfate pathway and thiosulfate pathway for L-cysteine synthesis. Proteins and genes involved in assimilation of inorganic sulfur sources in *E. coli* are shown. APS, adenosine 5-phosphosulfate; PAPS, 3-phosphoadenosine 5-phosphosulfate; OAS, *O*-acetylserine; GSG, glutathione.

Because high levels of L-cysteine have been reported to be toxic to cells [2,3], intracellular L-cysteine level is strictly controlled. Excess L-cysteine inhibits the activity of L-serine *O*-acetyltransferase (SAT), a key enzyme in the L-cysteine biosynthetic pathway (Figure 1) [4]. We have so far constructed recombinant *E. coli* strain that overproduces L-cysteine/L-cystine by expressing feedback inhibition-insensitive SAT [5]. The L-cysteine production has been also improved by disrupting L-cysteine desulfhydrases (CD). Five different CDs (MetC, MalY, CysK, CysM, and TnaA), which degrade to pyruvate, ammonia, and sulfide from L-cysteine, have been characterized in *E. coli*[6]. In addition, overexpression of L-cysteine exporters (YdeD, YfiK, Bcr, and TolC) significantly increased L-cysteine production, probably due to the decrease in cellular L-cysteine level [7-9]. For further improvements in L-cysteine production, one can consider enhancement of the downstream pathway of SAT, which includes incorporation of sulfur into OAS.

In Gram-negative bacteria, *E. coli* and *Salmonella typhimurium*, the sulfate-thiosulfate permease is a complex of five types of subunits encoded by: *sbp*, *cysP*, *cysU*, *cysW*, and *cysA* genes [10]. Sulfate assimilation is initiated by periplasmic sulfate (Sbp) and thiosulfate (CysP) binding proteins [11]. *E. coli* has two enzymes which assimilate inorganic sulfur sources, CysK and CysM. While the former enzyme utilizes sulfide (S^2-^) as a sulfur donor, the latter enzyme uses thiosulfate (SSO_3_^2-^). CysK synthesizes L-cysteine from *O*-acetylserine (OAS) and sulfide, but the CysM protein differs in that it can also utilize thiosulfate instead of sulfide (Figure 1). The product formed by the CysM activity, *S*-sulfocysteine (SSC), is converted into L-cysteine and sulfite by an uncharacterized reaction [12].

Sulfite is an intermediate of the sulfate pathway. The *E. coli* sulfite reductase consists of the alpha subunit protein (the *cysJ* gene product) and the beta subunit protein (the *cysI* gene product). The sulfate pathway spends two molecules of ATP and four molecules of NADPH as a reducing power to make L-cysteine from sulfate and OAS. On the other hand, the thiosulfate pathway has the advantage that two molecules of L-cysteine can synthesize from a thiosulfate molecule by consuming only one molecule of NADPH.

As thioredoxins (Trxs) and glutaredoxins (Grxs) are known as reductases of peptidyl disulfides [13], we expected that overexpression of these reductases might contribute to improving L-cysteine production by accelerating the reduction of SSC in *E. coli*. In this study, we identified the reductases involved in the reduction of SSC in the thiosulfate pathway and examined the effect of such reductases on L-cysteine production in *E. coli*.

## Results

### Trxs and Grxs can reduce SSC to L-cysteine *in vitro*

To eliminate the possibility of reduction of non-enzymatic SSC by GSH, we investigated first about the influence on cystein production by the Δ*gshA* strain, which cannot produce GSH, in thiosulfate as a sole sulfur source. The Δ*gshA* of L-cysteine overproducer increased L-cysteine production (Additional file 1), suggesting that GSH is not essential for reduceing SSC *in vivo*.

**Figure 2:**
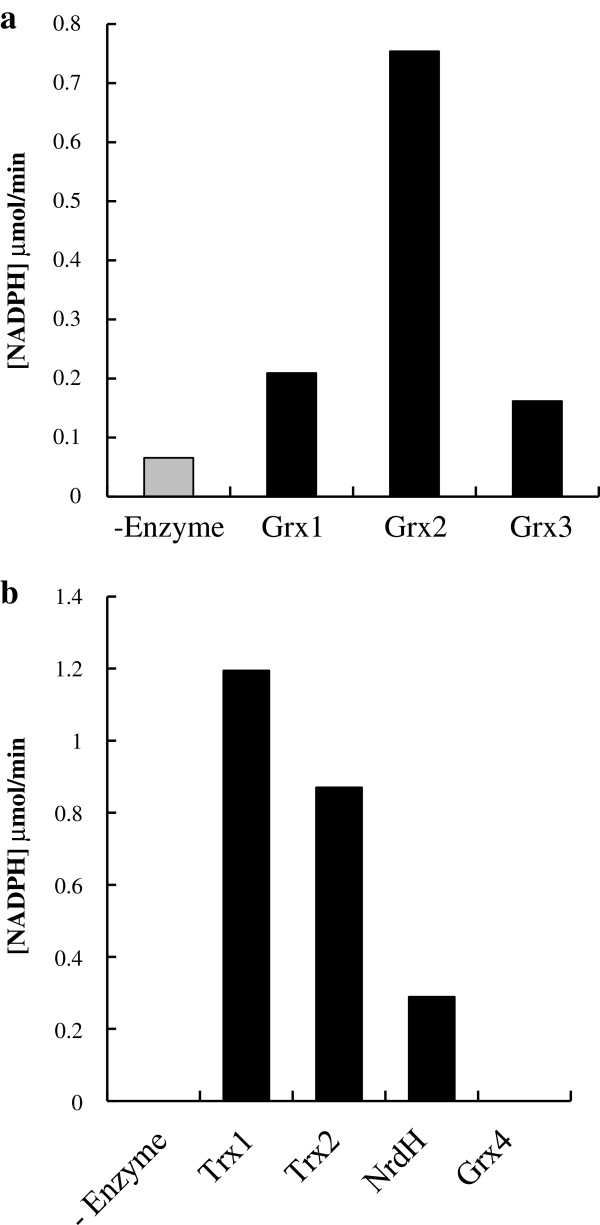
**Reduction of SSC to L-cysteine by Trxs and Grxs *in vitro.*** (**a**) Reducing activity of SSC by Grxs was measured in a reaction mix containing NADPH, GSH, GSH reductase and SSC by monitoring the decrease of NADPH, when adding 10 pmoles of purified Grxs (Grx1-3) for 3 minutes. (**b**) Reducing activity of SSC by Trxs was measured in a reaction mix containing NADPH, Trx reductase and SSC by monitoring the decrease in absorbance of NADPH at 340 nm for 3 minutes, when adding 10 pmoles of purified Trxs (Trx1-2, NrdH and Grx4).

It has been reported that *E. coli* has two Trxs (Trx1 and Trx2), three Grxs (Grx1, Grx2, and Grx3) and two Grx-like proteins (Grx4 and NrdH). Since the redox enzymes can reduce the disulfide that forms on proteins, we first tested whether these enzymes, catalyze the reduction of SSC to L-cysteine *in vitro* (Figure 2). The His-tagged recombinant protein of each enzyme was purified with a His-trap column as described in the Methods section. The SSC-reducing activity was determined by monitoring the decrease in absorbance of NADPH at 340 nm. The reaction mixture for Trx assay contains NADPH-dependent thioredoxin reductase to reduce Trxs [14]. On the other hand, since the Grx assay mixture contains glutathione (GSH) as well as GSH reductase, the feeble NADPH oxidation activity was detected in the absence of Grx [15], suggesting that GSH has a weak reduction activity of SSC. However, in the presence of Grx, the activity was significantly increased (Figure 2a). Among all redox enzymes, we found that the reducing activities of Grx2, Trx1, and Trx2 were higher than that of other proteins. No activity was observed in Grx4 (Figure 2b). It should be noted that L-cysteine, which is a reaction product from SSC, was also detected in the reaction mixture (data not shown).

### Overexpression of Trxs or Grxs improves L-cysteine productivity *in vivo*

**Figure 3:**
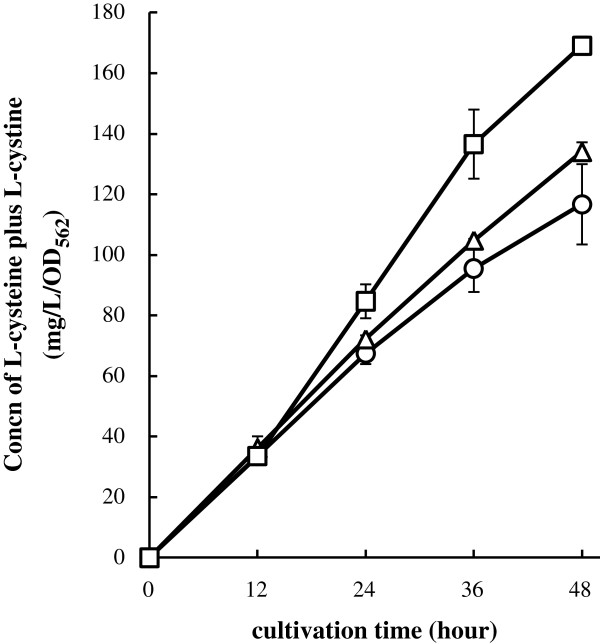
**Improvement of L-cystine production by NrdH or Grx1 *in vivo. ***L-cysteine plus L-cystine production by L-cysteine producer with plasmid overproducing Grx1 or NrdH genes. BW25113 harboring the L-cysteine over producing plasmid pDES (L-cysteine overproducer) was transformed empty vector pCA24N (*circles*), pGrx1 (*triangles*) or pNrdH (*squares*). These strains were cultivated in SM1 medium with 30 mM thiosulfate for 48 hours. The concentration of L-cysteine plus L-cystine was determined by Gaitonde method. Values indicate means and standard deviations of results from three independent experiments.

We further examined whether these redox enzymes contribute to improving the L-cysteine productivity. We previously found that there was a significant production of L-cysteine from glucose in *E. coli* cells carrying the plasmid pDES, which includes the wild-type *ydeD* gene and the altered genes of *cysE* and *serA*[9]. The above *E. coli* strain harboring pDES was designated as a L-cysteine producer [9]. Growth of the L-cysteine producers overexpressing each redox enzyme was the same level (data not shown). We also determined L-cysteine plus L-cystine level in culture medium of L-cysteine producers (Figure 3). After 48 h of cultivation, L-cysteine producer cells overexpressing Grx1 or NrdH showed a higher amount of L-cysteine than that of cell carrying the empty vector. However, overexpression of Trx1, Trx2, Grx2, and Grx3 did not improve L-cysteine productivity (Additional file 2). These results indicate that overexpression of either Grx1 or NrdH is effective for improving the L-cysteine productivity.

### Grx1 or NrdH converts SSC into L-cysteine in *E. Coli* under L-cysteine production

**Figure 4:**
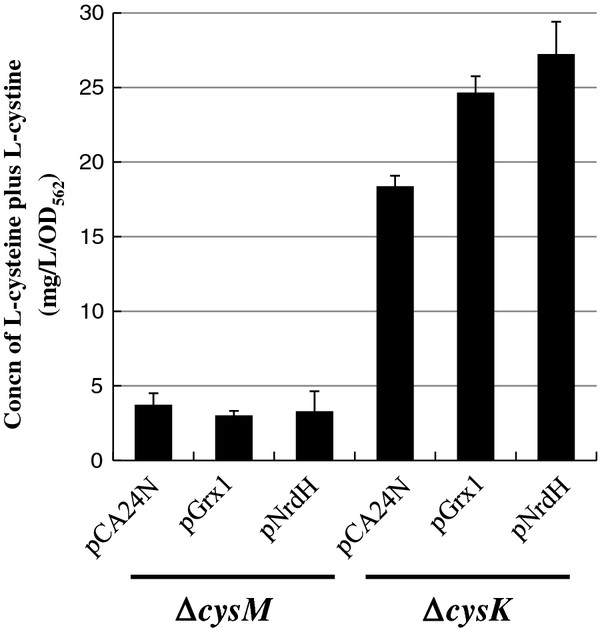
**CysM dependent L-cysteine production.** Δ*cysM* or Δ*cysK* harboring pDES was transformed plasmids overexpressing empty vector (pCA24N), pGrx1 or pNrdH. These strains were cultivated in SM1 with 30 mM thiosulfate and 30 mM sulfate medium for 36 hours to compare amount of L-cysteine produced from Δ*cysM* or Δ*cysK*, respectively. The concentration of L-cysteine plus L-cystine was determined by Gaitonde method. Values indicate means and standard deviations of results from three independent experiments.

As shown in Figure 4, in Δ*cysK* cells carrying pDES, overexpression of Grx1 or NrdH significantly enhanced L-cysteine production than that of cells carrying the empty vector after 36 h of cultivation. In contrast, when Grx1 or NrdH was overexpressed, there was no significant change in L-cysteine production in Δ*cysM* cells carrying pDES (Figure 4). These results strongly suggest that Grx1 or NrdH is involved in L-cysteine production from SSC, which is synthesized from thiosulfate and OAS by CysM. Furthermore, Δ*cysM* cells hardly produced L-cysteine even in the presence of sulfate as well as thiosulfate.

### Sulfite produced from the SSC reduction is utilized to produce L-cysteine in the sulfate pathway

**Figure 5:**
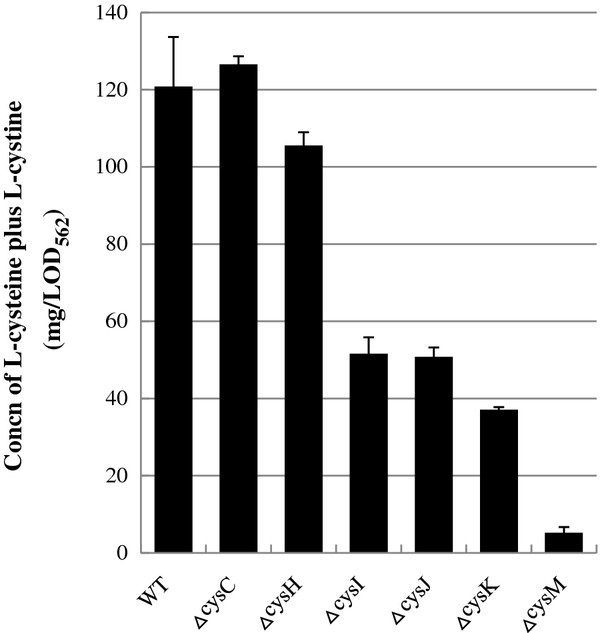
**Incorporation of sulfite generated from SSC reduction into the sulfate pathway to produce another L-cysteine.** Wild type, Δ*cysC*, Δ*cysH*, Δ*cysG*, Δ*cysI*, Δ*cysJ*, Δ*cysK*, or Δ*cysM* harboring pDES were cultivated in SM1 with 30 mM thiosulfate for 36 hours. The concentration of L-cysteine plus L-cystine was determined by Gaitonde method. Values indicate means and standard deviations of results from three independent experiments.

In addition to L-cysteine, sulfite is produced through the SSC reduction. It appears that sulfite is another sulfur source, as being on intermediate of the sulfate pathway. To address this possibility, we analyzed the effect of lack of the genes involved in the sulfate pathway on L-cysteine production (Figure 5). As expected, Δ*cysI*, Δ*cysJ,* or Δ*cysK* cells harboring pDES each showed to about 50% decrease in L-cysteine production compared to that observed in wild-type cells. Interestingly, its yield in Δ*cysC* or Δ*cysH* was equivalent the same as that of wild-type cells. These results clearly indicate that overexpression of NrdH or Grx1 in L-cysteine producer converts SSC into L-cysteine and sulfite. It was also shown that generated sulfite is utilized as a sulfur source to produce additional L-cysteine molecule through the sulfate pathway.

### Co-overexpression of CysI, CysK and NrdH confers the increased L-cysteine productivity on *E. Coli* cells

**Figure 6:**
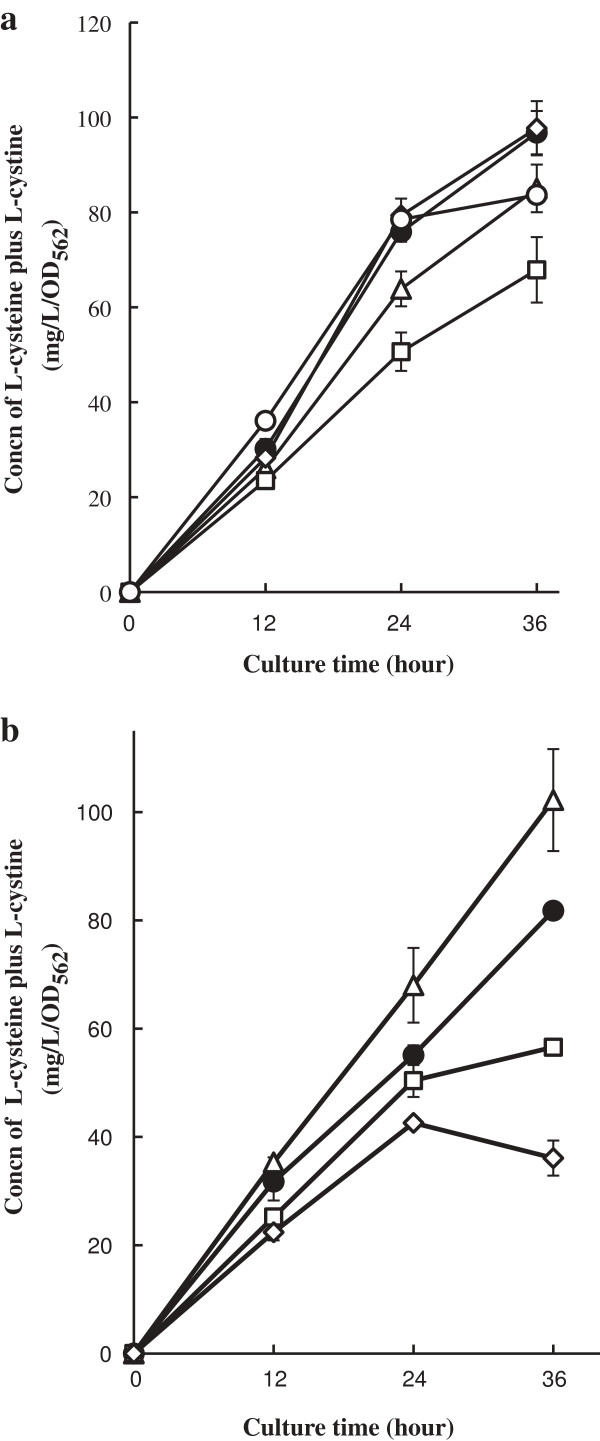
**Effect of L-cysteine production by enhancement of the sulfite reductases and/or the L-cysteine synthases.** L-cysteine overproducer was transformed (**a**) empty vector pCA24N (*closed circles*), pCysI (*opened triangles*), pCysJ(*opened squares*), pCysG (*opened diamonds*) and pCysK (*opened circles*), (**b**) pCA24N (*closed circles*), pCysI-K (*opened triangles*), pCysJ-K (*opened squares*) and pCysI-J (*opened diamonds*) to promote the incorporation of sulfite reduced from SSC to the sulfate pathway. These strains were cultivated in SM1 medium with 30 mM thiosulfate for 36 hours. The concentration of L-cysteine plus L-cystine was determined by Gaitonde method. Values indicate means and standard deviations of results from three independent experiments.

**Figure 7:**
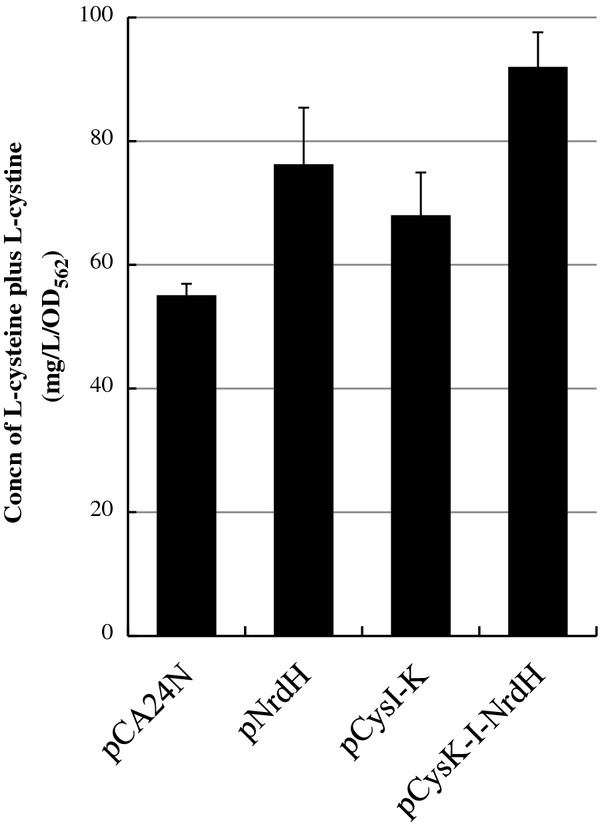
**Increase in L-cysteine production by the co-overexpression of CysI-CysK-NrdH.** These strains were cultivated in SM1 medium with 30 mM thiosulfatens and standard deviations of results from three independent experiments.

The results shown in Figure 5 led us to strengthen the reuse of sulfite pathway in L-cysteine synthesis. To improve L-cysteine productivity, we first attempted overexpression of sulfite reductase (CysI and CysJ) or L-cysteine synthase (CysK). The middle-copy-numbered plasmids pCysI, pCysJ, or pCysK was additionally transformed into L-cysteine producer cells. The transformants were cultivated in SM1 medium containing thiosulfate as a sole sulfur source. As shown in Figure 6a, overexpression of either CysI, CysJ, or CysK did not lead to an increase production of L-cysteine. Next, we evaluated L-cysteine productivity by co-overexpressing both sulfite reductase and L-cysteine synthase. The L-cysteine productivity in L-cysteine producer cells carrying pCysI-J or pCysJ-K was lower than that of cells harboring pCA24N (Figure 6b). On the other hand, overexpression of CysI and CysK increased L-cystine production. Finally, we found that the productivity of L-cysteine producer cells co-overexpressing CysI, CysK, and NrdH was significantly higher than that of cells overexpressing only NrdH or CysI and CysK after 24 h of cultivation (Figure 7). In addition to expression of feedback-insensitive SAT, our results concluded that the enhancement of L-cysteine biosynthesis is achieved by (i) utilization of thiosulfate as a sulfur source, (ii) overexpression of SSC reductases, and (iii) acceleration of sulfite reduction.

## Discussion

*Escherichia coli* has two L-cysteine biosynthetic pathways: one is synthesized from OAS and sulfate by CysK (the sulfate pathway), and the other is synthesized via SSC from OAS and thiosulfate by CysM (the thiosulfate pathway) (Figure 1). Indeed, Δ*cysM*Δ*cysK* cells exhibited a L-cysteine auxotrophy (Additional file 3). The genes involved in L-cysteine biosynthesis and sulfur assimilation in *E. coli* and *Salmonella enterica* serovar Typhimurium have been well characterized [4]. However, the conversion of SSC to L-cysteine remained unclear in these organisms. SSC has been believed to non-enzymatically convert into L-cysteine and sulfite by GSH *in vivo*[16]. However, a defect in the glutathione biosynthesis (Δ*gshA*) of L-cysteine overproducer increased L-cysteine production, suggesting that GSH is not essential for reduceing SSC *in vivo* (Additional file 1). Therefore, we first focused on redox enzymes of *E. coli*, especially Trxs and Grxs, to accelerate the reduction of SSC. Our results clearly indicate that recombinant redox enzymes (Trx1, Trx2, Grx1, Grx2, Grx3, and NrdH) reduce SSC to L-cysteine more effectively than a reducing agent such as GSH *in vitro* (Figure 2). Moreover, Grx2 had the highest SSC-reducing activity (Figure 2a). This result is consistent with the evidence that Grx2 has higher catalytic activity to a low molecular weight substrate such as HED compared to Grx1 and Grx3 [17]. *In vivo*, on the other hand, overexpression of NrdH or Grx1 is effective for L-cysteine production (Figure 3). In fact, the L-cysteine producer/pGrx1 or pNrdH produced about 15% or 45% higher amounts of L-cysteine, respectively, compared to the parental strain (Figure 3). Unexpectedly, overexpression of Grx2, Grx3, Trx1, and Trx2 were not effective for L-cysteine production (Additional file 2), suggesting that these enzymes might not reduce low molecular disulfides like oxidized glutathione, cystine, and SSC *in vivo*. It has hardly been reported so far that all redox enzymes except for Grx2 reduce low molecular disulfides *in vivo*. Moreover, as shown in Figure 4, the improvement of L-cysteine productivity by overexpression of Grx1 and NrdH depends on that of CysM. The present report is the first finding to show that Grx1 and NrdH release sulfite from SSC to produce L-cysteine under the fermentative conditions. Next, we challenged acceleration of sulfite utilization. Our results obviously indicate that the sulfite from the reduction of SSC is recruited into the sulfate pathway and is utilized for L-cysteine synthesis under L-cysteine fermentation (Figure 5). Consistent with our speculation, the CysI-K and NrdH co-overexpression in L-cysteine overproducer showed the highest L-cysteine productivity (Figures 6 and 7), suggesting that assimilation of the SSC-mediated sulfite, but not the reduction of SSC, is a rate-limiting step. Since sulfite reductase CysJI forms a complex of CysJ and CysI proteins, the co-overexpression of *cysJ*, *cysI* and *cysK* may be more effective to increase the L-cysteine productivity than that of *cysI* and *cysK*. Interestingly, as shown in Figure 4 and 5, the Δ*cysM* cells harboring only pDES also hardly produced L-cysteine in the presence of sulfate as well as thiosulfate. This is the first report to indicate that the thiosulfate rather than sulfate is preference sulfur source for L-cysteine fermentation of *E. coli*. Therefore, *E. coli* cells might have a regulation system that synthesizes mainly L-cysteine from the energetically-favored thiosulfate, as the assimilation of sulfate spends two molecules of ATP and four molecules of NADPH.

We found that SSC (Minimum Inhibitory Concentration value [MIC], 80.5 mg/ml) as well as GSH (MIC, >20.1 mg/ml) is not toxic to cells different from L-cysteine (MIC, 2.2 mg/ml). Thus, SSC might play a role in the L-cysteine pool. It was recently reported that SSC and *S*-sulfocysteine synthase, which is a homolog of CysM, each play an essential roles in light-dependent redox regulation in plant chloroplasts [18]. Considering the previous and present findings, we suggest that *E. coli* cells have a regulatory system at the transcriptional level (CysB: activator of genes encoding the sulfate or thiosulfate uptake complex upon sulfur starvation) or at the protein level (CysP: thiosulfate binding protein) to utilize predominantly thiosulfate. Our findings show that the ability of thiosulfate can promote the fermentative production of the other organic sulfur-compounds, such as methionine, GSH, thiamine, taurine, and biotin, which are also commercially needed.

## Conclusions

Grx1 or NrdH indeed converts SSC into L-cysteine and sulfite in *E. coli* cells under fermentative conditions. We also provide evidence that sulfite generates by the SSC reduction through the sulfate pathway. In addition, co-overexpression of NrdH, CysI, and CysK increases L-cysteine production. Based on these results, we propose the enhancement of thiosulfate utilization as a novel method for improving L-cysteine production in *E. coli*.

## Methods

### Strains and plasmids

The *E. coli* strains and plasmids used in this study are listed in Table 1. Plasmid pDES (supplied by Ajinomoto) for the overproduction of L-cysteine, which carries tetracyclin resistance gene, is a derivative of pACYC184 containing the altered *cysE* gene encoding the L-cysteine feedback inhibition-insensitive mutant SAT (Thr167Ala), the wild-type *ydeD* gene encoding inner membrane L-cysteine transporter [7], and the altered *serA* gene encoding the L-serine feedback inhibition-insensitive mutant D-3-phosphoglycerate dehydrogenase (Thr410stop). Each gene fragment is under control of the constitutive promoter of the *E. coli ompA* gene encoding outer membrane protein A precursor. The altered *serA* gene is oriented in the same direction, the altered *cysE* and *ydeD* genes are in the opposite direction of replication origin, as described previously [9,19].

**Table 1:** Bacterial strains and plasmids used

**E. coli strain or plasmid**	**Genotype**	**Reference or source**
**Strains**		
BW25113	wild type	[21]
JW2720	*cysC*::Km^R^	[21]
JW3582	*cysE*::Km^R^	[21]
JW3331	*cysG*::Km^R^	[21]
JW2732	*cysH*::Km^R^	[21]
JW2733	*cysI*::Km^R^	[21]
JW2734	*cysJ*::Km^R^	[21]
JW2407	*cysK*::Km^R^	[21]
JW2414	*cysM*::Km^R^	[21]
AG1	$recA1\;endA1\;gyrA96\;thi-1\;hsdR17\left(r_{K}{-m}_{K}{}^{+}\right)\sup E44\;relA1$	[22]
**Plasmids**		
pDES	pACYC184 with *serA* (T410 Stop), *ydeD* and alterd *cysE* (T167A) genes under the control of the OmpA promoter	[9]
pCA24N	Cm^R^	[22]
pCysH	pCA24N, *cysH* gene on 0.7 kb DNA fragment	[22]
pCysI	pCA24N, *cysI* gene on 1.7 kb DNA fragment	[22]
pCysJ	pCA24N, *cysJ* gene on 1.8 kb DNA fragment	[22]
pCysK	pCA24N, *cysK* gene on 0.9 kb DNA fragment	[22]
pCysM	pCA24N, *cysM* gene on 0.9 kb DNA fragment	[22]
pGrx1	pCA24N, *grxA* gene on 0.26 kb DNA fragment	[22]
pGrx2	pCA24N, *grxB* gene on 0.62 kb DNA fragment	[22]
pGrx3	pCA24N, *grxC* gene on 0.25 kb DNA fragment	[22]
pGrx4	pCA24N, *grxD* gene on 0.35 kb DNA fragment	[22]
pTrx1	pCA24N, trxA gene on 0.33 kb DNA fragment	[22]
pTrx2	pCA24N, *trxC* gene on 0.42 kb DNA fragment	[22]
pNrdH	pCA24N, *nrdH* gene on 0.25 kb DNA fragment	[22]
pCysK-I	pCA24N, *cysK, cysI* gene on 2.6 kb DNA fragment	This study
pCysK-J	pCA24N, *cysK, cysIJ*gene on 2.7 kb DNA fragment	This study
pCysJ-I	pCA24N, *cysJ, cysI* gene on 3.5 kb DNA fragment	This study
pCysK-I-NrdH	pCA24N, *cysK*, *cysI*, *nrdH* gene on 3.0 kb DNA fragment	This study

To obtain the *cysI* or *cysJ* gene, a polymerase chain reaction (PCR) was performed with a set of primers 5’-TTC GTC GCG GCC GCG AAA TCA TAA AAA ATT -3’ and 5’- AAC AAT CCA GAT GAG TTC TGA -3’ (the underlining indicates the position of *Not*I) with the plasmid pCysI or pCysJ as a template, respectively. These PCR products was digested with *Not*I and subsequently cloned into pCysK or pCysI to give pCysK-I, pCysK-J, or pCysI-J. In addition, the *nrdH* gene was also amplified using a set of primers 5’- CGT CTT CAC CTG CGA GAA ATC ATA AAA AAT T -3’ and 5’- TAT CAA CTC GAG TCC AAG CTC AGC TAA TTA -3’(the underlining indicates the position of *Xho*I) with the plasmid pNrdH as a template. The PCR product was digested with *Xho*I and then ligated to the plasmid pCysK-I to construct pCysK-I-NrdH.

### Media and cultivation

Unless otherwise stated, Lennox (L) broth [1% Bacto Tryptone (Difco Laboratories, Detroit, Mich.), 0.5% Bacto Yeast Extract (Difco), and 0.5% NaCl], Luria-Bertani (LB) medium (1% Bacto Tryptone, 0.5% Bacto Yeast Extract, and 1% NaCl), and SM1 minimal medium (100 mM potassium phosphate buffer [pH 7.0], 150 mM NH_4_Cl, 1.7 mM NaCl, 1.0 mM MgCl, 0.1 mM CaCl_2_, 7.2 μM FeSO_4_, 3.4 mM trisodium citrate, 0.6 μM Na_2_MoO_4_, 40.4 μM H_3_BO_3_, 2.9 μM CoCl_2_, 1 μM CuSO_4_, 8.1 μM MnCl_2_, 1 μM ZnSO_4_, and 3.0% glucose (wt/vol) as the carbon source) were used for the general cultivations. If necessary, chloramphenicol (Cm; 30 μg/ml), kanamycin (Km; 50 μg/ml), or/and tetracycline (Tet; 10 μg/ml) was added. For solid media, 1.5% (wt/vol) agar was added. Where indicated, SM1 was supplemented with L broth (final 10%), l-methionine, and sulfur sources (sulfate: MgSO_4_ or/and thiosulfate: Na_2_S_2_O_3_). Cultures were incubated aerobically by vigorous shaking at 30°C or 37°C. Growth was monitored by measurement of the optical density at 660 nm (OD_660_).

In L-cysteine production experiment, SM1 medium with 30 mM thiosulfate and 30 mM sulfate or only 30 mM thiosulfate was used for the L-cysteine production. L-Cysteine overproducers were precultured in LB (Tet + Cm) at 30°C for 20 hours and the preculture was inoculated into 30 ml of SM1 + sulfur source (Tet + Cm) to set into OD_660_ of 0.4 in each culture. The culture was cultivated at 30°C for 24–48 h. Isopropyl-1-thio-β-d-galactopyranoside (IPTG; final concentration 0.1 mM) was added to the medium after 6 hours cultivation to overproduce the intended proteins. The pH was adjusted to 7.0 using CaCO_3_. Growth was measured by absorbance (OD_562_) of culture broth after appropriate dilution with 0.1 N HCl.

### Expression and purification of recombinant proteins

*E. coli* K-12 strain AG1 [*recA1 endA1 gyrA96 thi-1 hsdR17* (r_K_^-^m_K_^+^) *supE44 relA1*] was transformed with each of the various pCA24N-based plasmids, and then the transformed cells were grown at 30°C in 50 ml of LB medium. When OD_660_ reached 0.6, IPTG was added to the culture medium to a final concentration 0.1 mM to induce gene expression. After cultivation for 3 h at 30°C, the cells were harvested, suspended in 5 ml of ice-cold buffer A (40 mM imidazole, 0.5 M NaCl, 20 mM Sodiun Phosphate), and broken by sonic oscillation under cooling. After centrifugation (20 min at 18,000 × *g*), the soluble fraction of the supernatant was purified using Ni sepharose column, His Trap HP(GE Healthcare, Piscataway, NJ) by the procedure recommended by the supplier. His-tagged fusion proteins were applied to the column equilibrated with the same buffer containing 10 mM imidazole. The column was washed with 80 mM imidazole in the same buffer, and proteins were eluted with 0.5 M imidazole, 0.5 M NaCl and 20 mM sodium phosphate in the same buffer. Protein concentrations were determined using a Bio-Rad protein assay kit (Hercules, CA) with bovine serum albumin as the standard protein.

### Enzyme assay

Trx and Grx activity was assayed by the procedures as described in [14,15]. Trxs (Trx1-2) and Grx-like proteins (Grx4 and NrdH) are rereduced by Trx reductase after the reduction of substrates. Grxs (Grx1-3) are also rereduced by glutathione and glutathione reductase. We therefore separately measured the activities of Trxs and Grxs by monitoring the decrease in absorbance of NADPH at 340 nm. The reaction mixture for Grx assay contained the following in a final volume of 0.5 ml; 0.5 mM GSH, 0.16 mM NADPH, 0.1 mg/ml BSA, 5.9 μg/ml glutathione reductase (Sigma-Aldrich, St. Louis, MO) in 0.1 M Tris–HCl (pH 8.0). The disulfide substrate, 50 μM *S*-sulfocysteine (Sigma-Aldrich), is added to the reaction mixture, leading to the formation of a mixed disulfide between GSH and *S*-sulfocysteine within 1 min. After the recombinant Grx is added to the reaction mixture, the decrease of NADPH was measured for five minutes.

The reaction mixture for Trx assay contained the following in a final volume of 0.5 ml; 0.32 mM NADPH, 20 mM *S*-sulfocysteine, 0.2 μM thioredoxin reductase in 0.1 M potassium phosphate/2 mM EDTA (pH 7.0). The recombinant Trx was added to the reaction mixture to start the reduction of *S*-sulfocysteine.

### Determination of L-cysteine content

The amount of L-cysteine in culture supernatants was determined according to the procedure of Gaitonde [20]. Before adding a Gaitonde reagent (250 mg ninhydrin dissolved in a mixture of 4 ml of HCl and 16 ml of acetic acid), L-cystine in the samples was reduced by incubation with 5 mM dithiothreitol in 100 mM Tris–HCl buffer (pH 8.6) for 10 min. The reaction products were diluted with 99.5% (vol/vol) ethanol prior to measurement of the absorbance at 560 nm.

## Competing interests

The authors declare that they have no competing interests.

## Authors’ contributions

TN, NW, SM and IO carried out the experimental studies. TN, IO and HT wrote the manuscript. IO conceived the project, co-supervised HT, and checked the data. All authors read and approved the final manuscript.

## Supplementary Material

Additional file 1 **L-cysteine plus L-cystine production in L-cysteine producer disrupted *gshA *gene.** Click here for file

Additional file 2 L-cysteine plus L-cystine production in L-cysteine producer overexpressing Grx2, Grx3, Trx1 or Trx2. Click here for file

Additional file 3 **Growth of *ΔcysKΔcysM *double knockout mutant on minimum medium.** Click here for file
