# Supplementary material for: Enhancement of thioredoxin/glutaredoxin-mediated L-cysteine synthesis from S-sulfocysteine increases L-cysteine production in Escherichia coli
Source: Microb Cell Fact. 2012 May 18;11:62. doi: 10.1186/1475-2859-11-62 (PMC3528435; doi:10.1186/1475-2859-11-62)
Supplement: Additional file 1 — L-cysteine plus L-cystine production in L-cysteine producer disrupted gshA gene. [file 1475-2859-11-62-S1.pptx]

## Slide 1
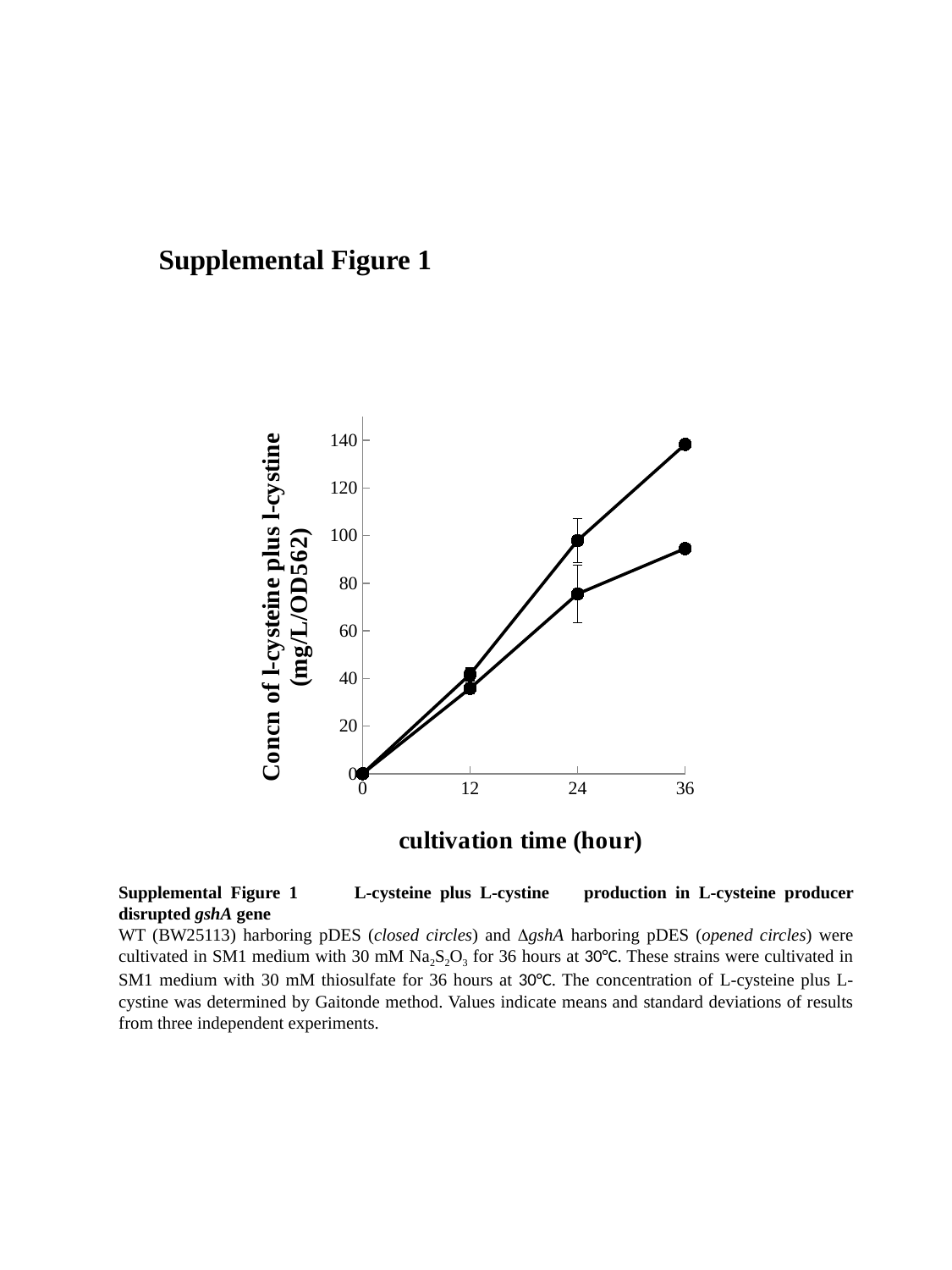

Supplemental Figure 1
### Chart
| Category | △gshA/pDES,pCA24N | WT/pDES,pCA24N |
|---|---|---|Supplemental Figure 1　　l-cysteine plus l-cystine　production in l-cysteine producer disrupted gshA gene
WT (BW25113) harboring pDES (closed circles) and DgshA harboring pDES (opened circles) were cultivated in SM1 medium with 30 mM Na2S2O3 for 36 hours at 30°C. These strains were cultivated in SM1 medium with 30 mM thiosulfate for 36 hours at 30°C. The concentration of l-cysteine plus l-cystine was determined by Gaitonde method. Values indicate means and standard deviations of results from three independent experiments.
