# Supplementary material for: Enhancement of thioredoxin/glutaredoxin-mediated L-cysteine synthesis from S-sulfocysteine increases L-cysteine production in Escherichia coli
Source: Microb Cell Fact. 2012 May 18;11:62. doi: 10.1186/1475-2859-11-62 (PMC3528435; doi:10.1186/1475-2859-11-62)
Supplement: Additional file 3 — Growth of ΔcysKΔcysM double knockout mutant on minimum medium. [file 1475-2859-11-62-S3.pptx]

## Slide 1
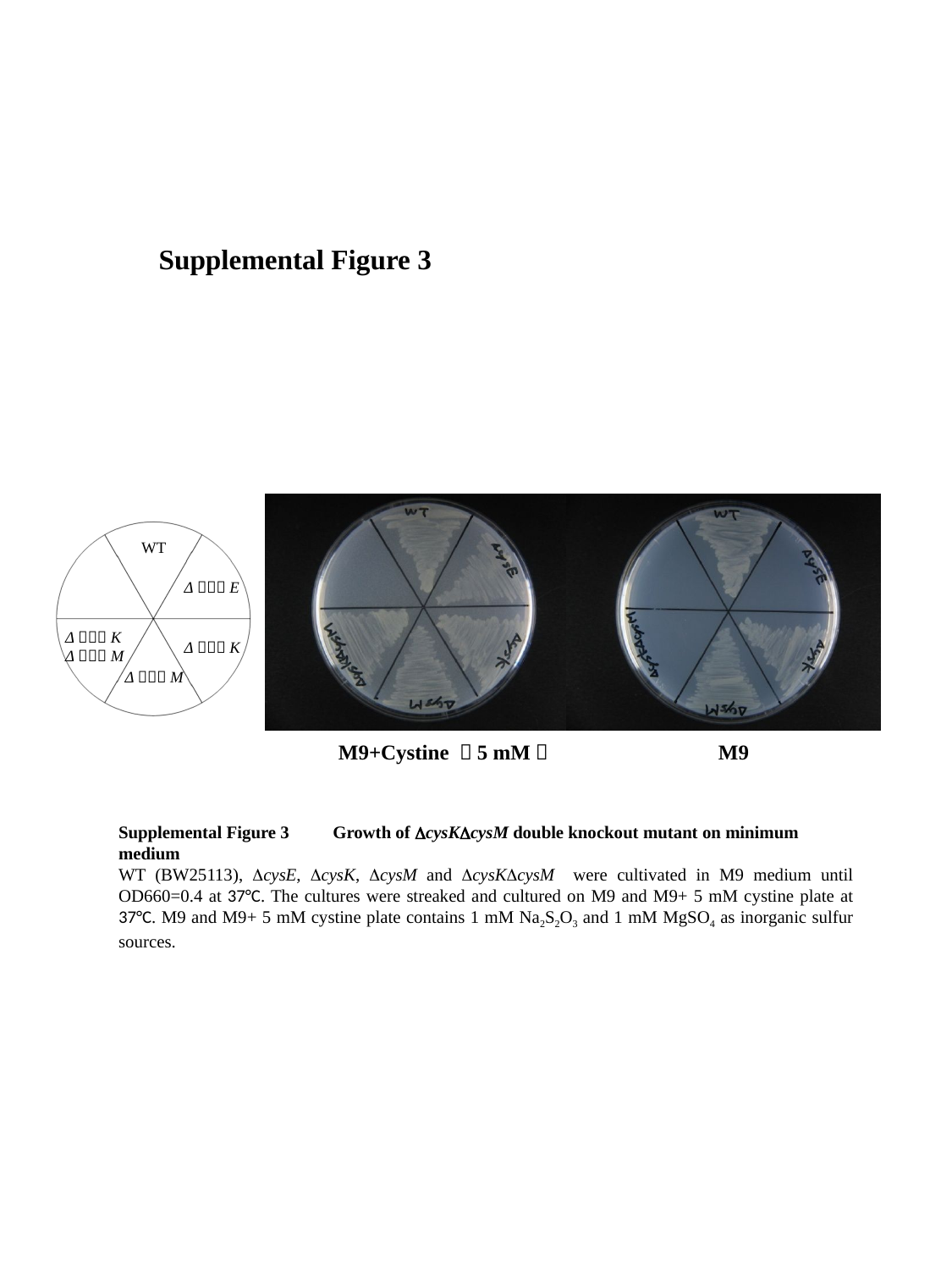

Supplemental Figure 3
WT
ΔｃｙｓE
ΔｃｙｓK
ΔｃｙｓM
ΔｃｙｓK
ΔｃｙｓM
M9+Cystine （5 mM）
M9
Supplemental Figure 3　　Growth of DcysKDcysM double knockout mutant on minimum medium
WT (BW25113), DcysE, DcysK, DcysM and DcysKDcysM were cultivated in M9 medium until OD660=0.4 at 37°C. The cultures were streaked and cultured on M9 and M9+ 5 mM cystine plate at 37°C. M9 and M9+ 5 mM cystine plate contains 1 mM Na2S2O3 and 1 mM MgSO4 as inorganic sulfur sources.
